# Stretchable Conductive Hybrid Films Consisting of Cubic Silsesquioxane-capped Polyurethane and Poly(3-hexylthiophene)

**DOI:** 10.3390/polym11071195

**Published:** 2019-07-17

**Authors:** Keigo Kato, Masayuki Gon, Kazuo Tanaka, Yoshiki Chujo

**Affiliations:** Department of Polymer Chemistry, Graduate School of Engineering, Kyoto University, Katsura, Nishikyo-ku, Kyoto 615-8510, Japan

**Keywords:** POSS, polyurethane, conducting polymer, organic–inorganic hybrid, stretchable electronics

## Abstract

We fabricated stretchable and electric conductive hybrids consisting of polyhedral oligomeric silsesquioxane (POSS)-capped polyurethane (PUPOSS) and doped poly(3-hexylthiophene) (P3HT). In order to realize robust films coexisting polar conductive components in hydrophobic elastic matrices, we employed POSS introduced into the terminals of the polyurethane chains as a compatibilizer. Through the simple mixing and drop-casting with the chloroform solutions containing doped P3HT and polyurethane polymers, homogeneous hybrid films were obtained. From the conductivity and mechanical measurements, it was indicated that hybrid materials consisting of PUPOSS and doped P3HT showed high conductivity and stretchability even with a small content of doped P3HT. From the mechanical studies, it was proposed that POSS promoted aggregation of doped P3HT in the films, and ordered structures should be involved in the aggregates. Efficient carrier transfer could occur through the POSS-inducible ordered structures in the aggregates.

## 1. Introduction

Stretchable electronics which show high performance and mechanical flexibility are recognized as a one of promising platform for realizing next-generation applications of optoelectronic devices such as deformable displays [1], electronic skins [2,3] and the property of mechanical invisibility for wearable [4,5] and implantable applications [6,7]. Since organic materials have various advantages as a scaffold, such as softness and ease of tuning and their electronic and mechanical properties, organic composites consisting of elastomeric polymers and semiconducting or conducting polymers have been developed as stretchable electronics [8,9,10,11,12,13,14,15,16,17,18,19,20,21,22]. For example, it has been reported that the nanoconfinement of semiconducting polymers into elastomers is valid for achieving high stretchability without decreasing carrier mobility [9]. In this system, high stretchability of elastomers and electronic conductivity of semiconducting polymers were simultaneously realized in the single material. Programmed phase separation should play a critical role in the compatibility of these properties at a high level. As another instance, it was shown that kirigami-based nanoconfined polymer sheets could be stretched up to 2000% strain without loss of carrier mobility [14]. To produce advanced stretchable optoelectronic devices, facile and versatile strategies for preparing materials are strongly desired.

Polyurethanes (PUs) are well known as an elastomer with property tunability. Therefore, PUs have been widely used in various fields, such as molds, paints, coating, foam, elastomer, etc. [23,24]. Additionally, PUs are also applied as a scaffold in composite materials in order to impart stretchability [25,26,27,28,29]. Meanwhile, stretchable composites consisting of elastomers and electrical conductive components, such as metal [30,31], carbon [32,33] and conducting polymers [34,35,36,37], have been manufactured for realizing stretchable electronics. Hydrophobic elastomers such as polydimethylsiloxanes (PDMS) are known as one of platforms, however there are often difficulties in preparing homogeneous materials with conductive polymers such as poly(3,4-ethylenedioxythiophene):poly(stylenesulfonate) (PEDOT:PSS) due to polarity differences between hydrophobic elastomers and hydrophilic doped conjugated polymers. In the case of PU, polar urethane structures are potentially favorable for improving compatibility. Indeed, composites were able to be obtained with PEDOT:PSS [18].

Conductive materials are prepared readily by chemical doping of conjugated polymers [38]. Owing to the discovery of conductive polyacetylene by Shirakawa et al. who brought development to the field of organic electronics [39], facile methods can be established for drastically improving conductivity of conjugated polymers by several orders of magnitude by doping [40]. So far, many researches have devoted their efforts to obtaining highly-efficient conjugated polymers, and it was revealed that a strong interchain interaction was responsible for smooth carrier transfer followed by high conductivity [41,42,43]. Poly(3-hexylthiophene) (P3HT) is one of the most famous semicrystalline conjugated polymers and has been extensively studied for the relationship between morphology and electronic properties [44,45,46,47,48]. Moreover, it was shown that regular structures were responsible for expressing high conductivity [44,45,46]. It should be noted that there are several reports regarding high conductive materials containing small molecules which can efficiently change the morphology of the polymers and subsequently lead to enhancement of the conductivity [47,48]. Thus, it is presumable that further functions except for conductivity might be added by their combination with other components.

Polyhedral oligomeric silsesquioxane (POSS) has attracted greater interest as an “element-block”, which is a minimum functional unit containing various types of elements, for obtaining hybrid materials simply by mixing [49,50]. POSS has high chemical and thermal stability, bulky and rigid hydrophobic space and high designability according to its unique structure [51,52,53,54,55,56,57,58,59,60,61,62]. For example, simple loading of POSS onto polymers with or without covalent bonding reinforces thermal stability and rigidity [63]. In particular, polymers containing POSS show unique properties depending on the morphology of polymer matrices [64,65,66,67]. In the previous work, we synthesized POSS-capped PU (PUPOSS) in which the hydrophobic POSS was covalently bound into the terminals of the polar PU chain [68]. The hydrophobicity of POSS improved compatibility with conjugated polymers, and stretchable polyfluorene (PF)/PUPOSS hybrids presenting high luminescence were achieved by suppressing the aggregation of PF without decreasing elasticity. Finally, formation of excimer was able to be controlled by mechanical forces to the elastic film, followed by emission color change.

On the basis of these results, we next aimed to load different functions, which tend to be in the trade-off relationship, onto PUPOSS-based elastic hybrids. We herein showed stretchable hybrid materials consisting of PUPOSS and doped P3HT possessing high conductivity even in a small content of doped P3HT. In the previous work, POSS suppressed the aggregation of conjugated polymers because of similarity in the polarity of POSS and PF [68], whereas, in this work, POSS promoted aggregation of doped P3HT because of a polarity difference between POSS and doped P3HT. As a result, electrical conductivity of doped P3HT/PUPOSS hybrid films was dramatically enhanced without significant losses of elasticity of the bulk films. The result indicates that POSS has the potential to work as a mediator to enable elastic and electric conductive moieties to independently exist in the materials without critical phase separation, which leads to deterioration of device performances.

## 2. Experimental Details

### 2.1. Materials

Compounds P3HT were synthesized according to the literature [69] 3-Aminopropyltrimethoxysilane, Methylenediphenile-4,4′-diisocyanate (MDI), dodecylamine, tetrafluoretetracyanoquinodimethane (F4-TCNQ) and dibutyltin Dilaurate (DBTDL) were purchased from Tokyo Chemical Industry Co., Ltd. (Tokyo, Japan) and used without further purification. 1,6-Hexanediol (HDO), poly(tetramethylene oxide) (PTMG), propylamine and hexane were purchased from Wako Pure Chemical Industries (Tokyo, Japan)and used without further purification. THF were purchased from Wako Pure Chemical Industries and were purified using a two-column solid-state purification system (Glasscontour System, Joerg Meyer, Irvine, CA, USA) under Ar pressure.

### 2.2. Synthesis and Characterization

**Scheme 1:**
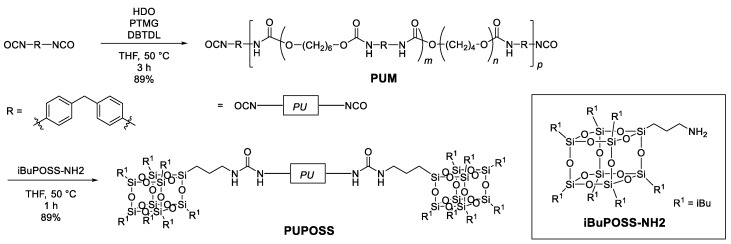
Synthesis of PUPOSS and PUM.

**Scheme 2:**
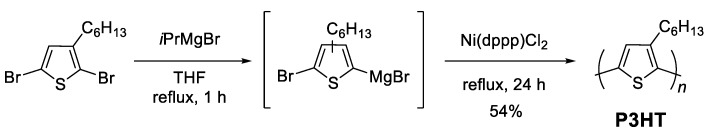
Synthesis of P3HT.

Scheme 1 shows the synthesis of PUPOSS and PUM. PUM was prepared through polyaddition of 1,6-hexanediol (HDO), poly(tetramethylene glycol) (PTMG) (HDO/PTMG = 1/1 mol/mol) and tolylene-2,4-diisocyanate (TDI). The excess amount of TDI was added for completely presenting isocyanate groups at the chain terminal. PTMG was introduced for increasing elasticity of the product. After the alcohol groups were completely reacted, PUM was isolated as a white solid by reprecipitation into hexane from the tetrahydrofuran (THF) solution. PUPOSS was synthesized by the connection with amine groups in aminopropylisobutyl POSS (iBuPOSS-NH_2_) and the terminal isocyanate groups of PUM. The relative molecular weights of PUM (*M*_n_ = 1.4 × 10^4^ g mol^−1^, *M*_w_/*M*_n_ = 2.8) and PUPOSS (*M*_n_ = 1.3 × 10^4^ g mol^−1^, *M*_w_/*M*_n_ = 2.5) were estimated by gel permeation chromatography (GPC) with polystyrene standards by using chloroform as an eluent. As a conjugated polymer, regioregular P3HT was synthesized through the Grignard metathesis (GRIM) polymerization according to the literature (Scheme 2) [69]. The regioregularity of P3HT was examined by ^1^H NMR spectroscopy. The α methylene protons of the alkyl group were able to be resolved by two different diads, head-to-tail (HT) and head-to-head (TT). The expanded ^1^H NMR spectrum in Figure S1 was indicative of 97% for the HT linkage. The relative molecular weight of P3HT (*M*_n_ = 4.7 × 10^4^ g mol^−1^, *M*_w_/*M*_n_ = 1.5) was also estimated by GPC with the same instrument and standard set. The structures of all new compounds were confirmed by ^1^H, ^13^C and ^29^Si NMR spectroscopy. The synthetic details are described in the Supplementary Materials.

The introduction ratio of POSS units to the terminals of PUPOSS was calculated from the ^1^H NMR spectrum and *M*_n_ of PUPOSS (Figure S2). It was shown that HDO and PTMG should be reacted with MDI because the hydroxymethyl protons of HDO and PTMG completely disappeared in the ^1^H NMR spectra (Figures S3–S6). The number of repeating units (*p*) was estimated to be approximately 8 from the division of *M*_n_ of PUPOSS by the molecular weight of the repeating unit. Subsequently, in the ^1^H NMR spectrum of PUPOSS, the integral value ratio (*H*_a_/*H*_b_ = 1.55) was calculated from the peak at the benzyl positions, which originated from the reacted MDI moieties (3.86 ppm) (*H*_a_) and the peak of the *iso*-butyl groups derived from the reacted iBuPOSS-NH2 (0.61–0.59 ppm) (*H*_b_). Hence, it was revealed that about 1.5 eq. POSS groups were modified into a single PU chain, (introduction rate = 75%).

^1^H, ^13^C and ^29^Si NMR spectra were recorded on JEOL EX400 and AL400 instruments (Tokyo, Japan) at 400, 100 and 80 MHz, respectively. Samples were analyzed in CDCl_3_, and the chemical shift values were expressed relative to Me_4_Si as an internal standard. Scanning electron microscopy (SEM) recorded on a HITACHI FE-SEM SU8220 (Tokyo, Japan). Energy dispersive X-ray spectrometry (EDX) was recorded on a EMAX Evolution X-MAX (80 mm^2^) (Tokyo, Japan). The number-average molecular weight (*M*_n_), the weight-average molecular weight (*M*_w_) and PDI (*M*_w_/*M*_n_) of all polymers were estimated by the gel permeation chromatography (GPC) with a TOSOH G3000HXI system (Tokyo, Japan) equipped with three consecutive polystyrene gel columns (TOSOH gels: a-4000, a-3000, and a-2500) and ultraviolet detector (Tokyo, Japan) at 40 °C. The system was operated at a flow rate of 1.0 mL/min, with chloroform as an eluent. Polystyrene standards were employed for the calibration. Electrical conductivity was recorded on a Mitsubishi Chemical LORESTA-EP (Tokyo, Japan). Differential scanning calorimetry (DSC) was recorded on a HITACHI DSC7020 (Tokyo, Japan). Thermogravimetric analysis (TGA) was recorded on a Seiko instrument Inc. EXSTAR TG/DTA6000 (Chiba, Japan). Dynamic mechanical analysis (DMA) was recorded on a SEIKO DMS210 (Chiba, Japan). The uniaxial tensile test was conducted at the speed of 1 mm/min by using an Orientec Corporation TENSILON RTM-500 (Tokyo, Japan).

### 2.3. Preparation of Hybrid Films

The film samples were prepared via the spin-coating on the quartz substrate with the chloroform solutions. The mixtures containing polyurethanes (PUPOSS or PUM) in CHCl_3_ (4 mL) were stirred at room temperature for 30 min. Then various amounts of P3HT (0–40 wt %) doped with F4-TCNQ (dopant molar fraction of 0.15) in CHCl_3_ (all solutions contained 1 mg of P3HT per 200 µL) were added to the mixtures and stirred at room temperature for an additional 1 h. The resulting solutions were dropped on the PFA dish and dried for 12 h under ambient condition to afford hybrid films, P3HT/PUPOSS and P3HT/PUM.

## 3. Results and Discussion

### 3.1. Dopant Concentration

We investigated the effect of dopant concentration on electric conductivity of P3HT films. As a dopant, we selected 2,3,5,6-tetrafluoro-7,7,8,8-tetracyano-quinodimethane (F4-TCNQ). Because of the deep energy level of LUMO, F4-TCNQ is known to be an effective dopant for P3HT (LUMO energy level of F4-TCNQ: −5.2 eV, HOMO energy level of P3HT: −5.0 eV) [70]. The measurement was carried out with the four probe method for evaluating in-plane conductivity of the films prepared by the drop-casting method from the chloroform solution (100 μL, 1.0 mg/200 μL P3HT) with different feed ratios of F4-TCNQ to P3HT on a quartz substrate (1 cm × 5 cm) [71]. The results showed that adding F4-TCNQ increased the conductivity of P3HT thin films up to a dopant molar fraction of 0.15 (Figure S7). As the optimized dopant concentration, this value was used in the following experiments.

### 3.2. Hybrid Materials

The hybrid materials consisting of PUM or PUPOSS and doped P3HT were prepared by the drop-casting method with the chloroform solutions on the perfluoroalkoxy alkane (PFA) dish. The detail preparation protocols are described in the Experimental Section. We obtained P3HT/PUPOSS and P3HT/PUM as a dark film (Figure S8). From scanning electron microscope (SEM) observations at the surface of the hybrid films (interface of hybrid film/PFA dish), different morphologies were obtained especially from the sample containing POSS. There were aggregates at the surfaces of each film, suggesting that polar doped P3HT species should be deposited (Figure S9). Significantly, in P3HT/PUPOSS, crystal-like particle aggregates were observed at the surface. This result proposes that POSS promoted aggregation formation with ordered structures. From the energy dispersive X-ray spectrometry (EDX) analyses at the cross-section of the hybrid films, homogeneous dispersion of Si from POSS and localization of S from P3HT toward the PFA side were observed (Figure S10). These data indicate that POSS should enhance to form aggregation of doped P3HT. Moreover, it was shown that the maximum amount of doped P3HT in P3HT/PUPOSS without loss of film-formability was 20 wt %, while the film sample was able to be obtained with 40 wt % doped P3HT in PUM. This supports the fact that the POSS moiety could promote the formation of aggregates of doped P3HT in the film. It was reported that dispersed POSS in the polymer matrix can act as a nucleation site to form aggregates of doped P3HT [67]. Moreover, it is likely that the polarity of the polymer matrix can be lowered in the presence of hydrophobic POSS. Thus, the aggregate formation of polar species, such as doped P3HT with F4-TCNQ, could be facilitated at the film surfaces.

### 3.3. Mechanical Properties

**Figure 1:**
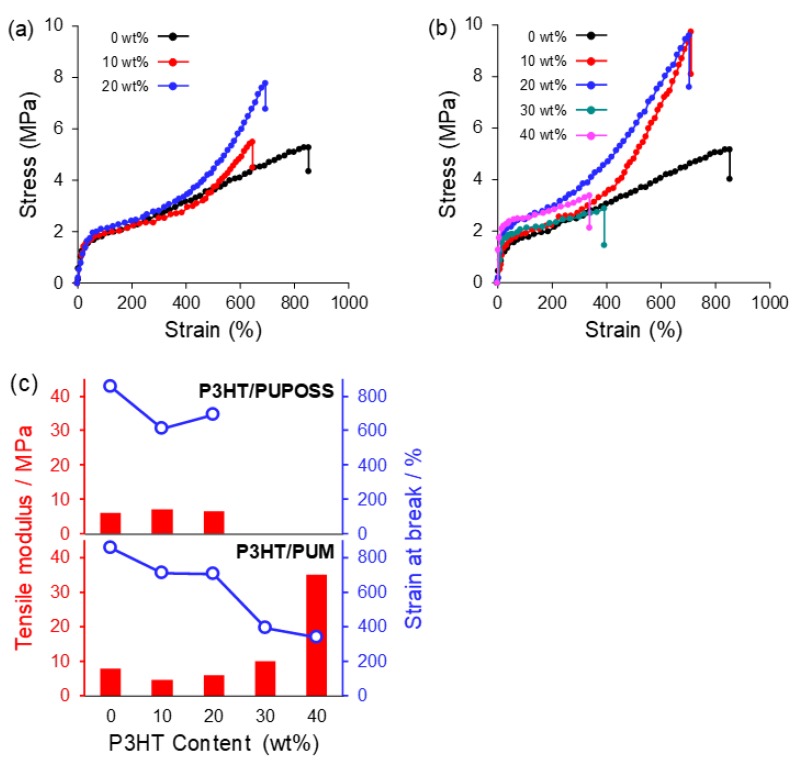
Stress-strain curves of (**a**) P3HT/PUPOSS and (**b**) P3HT/PUM at each amount of loaded doped P3HT. (**c**) Tensile moduli and strain values at the film breaking with P3HT/PUPOSS and P3HT/PUM.

Mechanical properties of the hybrid films were examined. The stress–strain relationships during the uniaxial extension were evaluated until the hybrid films were broken off by stretching (Figure 1 and Table 1). It should be emphasized that in P3HT/PUPOSS, the strain value at the break of the film retained high and the tensile modulus maintained low with the film containing 20 wt % of doped P3HT. These data clearly indicate that the stretchability of PUPOSS was hardly spoiled by mixing doped P3HT. As mentioned above, POSS modification induces aggregation formation of doped P3HT in the matrix, resulting in loss of film-formability over 30 wt %. On the other hand, critical decrease in mechanical properties was suppressed. In P3HT/PUM, the stretchability was maintained when loading up to 20 wt % of P3HT. However, when loading more than 30 wt % of P3HT, the strain value at the break of the film greatly dropped off and tensile modulus became much higher. These results indicate that critical loss of stretchability should occur. From dynamic mechanical analyses (DMA), it was shown that glass transition temperatures (*T*_g_) of both films decreased at 10 wt % P3HT loading ratio (Figure S11 and Table 1). By adding a further amount of doped P3HT, the *T*_g_ values elevated when increasing the content of doped P3HT. Especially, the *T*_g_ of P3HT/PUM exceeded room temperature when loading 30 wt % of P3HT. These results imply that the rubber state changes to the glass state at room temperature, leading to a decrease in stretchability although film-formability was obtained. Generally, aggregated domains tend to increase in tensile moduli compared to homogeneous hybridization. However, the increase could be too small to reduce elasticity in this material. It is proposed that POSS could work as a mediator and prevent critical phase separation which leads to deterioration of elasticity. Generally, aggregated domains tend to increase in tensile moduli compared to homogeneous hybridization. However, the increase could be too small to reduce elasticity in this material. It is proposed that POSS could work as a mediator and prevent critical phase separation which leads to deterioration of elasticity.

### 3.4. Aggregated Structure of P3HT

**Figure 2:**
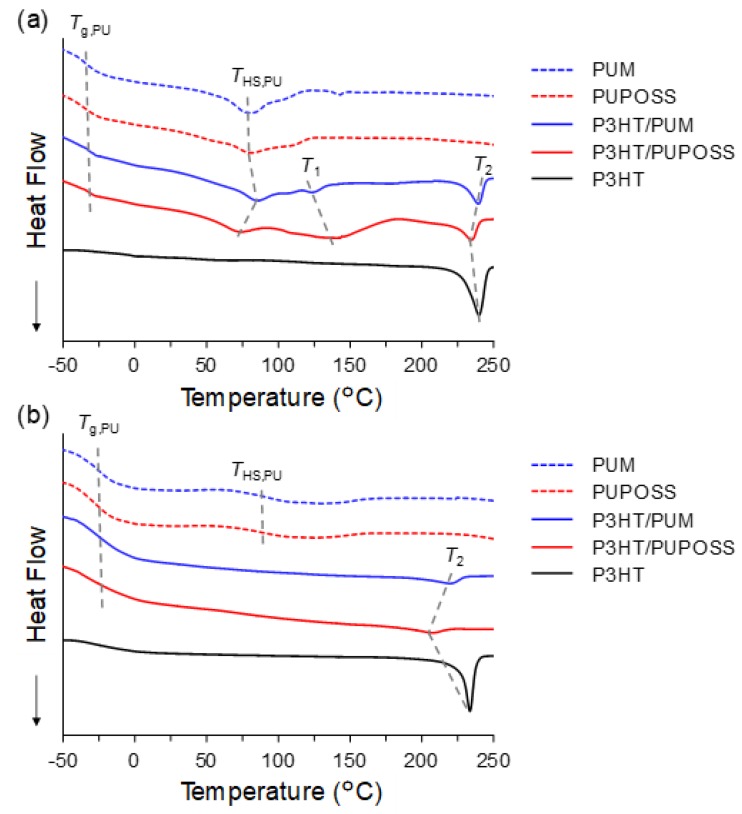
Differential scanning calorimetry curves of each film at (**a**) first and (**b**) second heating cycles. Heating rate was 10 °C/min. The content of P3HT of P3HT/PUM and P3HT/PUPOSS was 20 wt %.

To investigate the P3HT aggregated states in the hybrid films, differential scanning calorimetry (DSC) was performed (Table 2). Figure 2a shows the DSC first heating curves at the heating rate of 10 °C/min. In the DSC curves of all films, the glass transition temperatures of PU (*T*_g,PU_) and the relaxation of hard segments of PU (*T*_HS,PU_) were observed at around −40 and 60 °C, respectively [72]. In the DSC curves of P3HT/PUM and P3HT/PUPOSS, two melting temperatures appeared at around 130 °C (*T*_1_) and 220 °C (*T*_2_). *T*_2_ was assigned to the crystallization of main chains of P3HT [73] and heat of fusion was almost the same value with and without POSS. In contrast, *T*_1_ attributable to the crystallization of alkyl side chains of P3HT [73] and heat of fusion increased from 0.53 without POSS to 3.42 mJ mg^−1^ with POSS. It is suggested that well-ordered structures of P3HT should be developed in the aggregates in P3HT/PUPOSS more than in P3HT/PUM. Figure 2b shows the DSC second heating curves and the peaks attributable to the crystallization of P3HT alkyl side chains of P3HT/PUM and P3HT/PUPOSS, which completely disappeared. This result implies that ordered-aggregated structures of P3HT might be kinetically produced and thermodynamically unstable.

### 3.5. Electrical Conductivity

**Figure 3:**
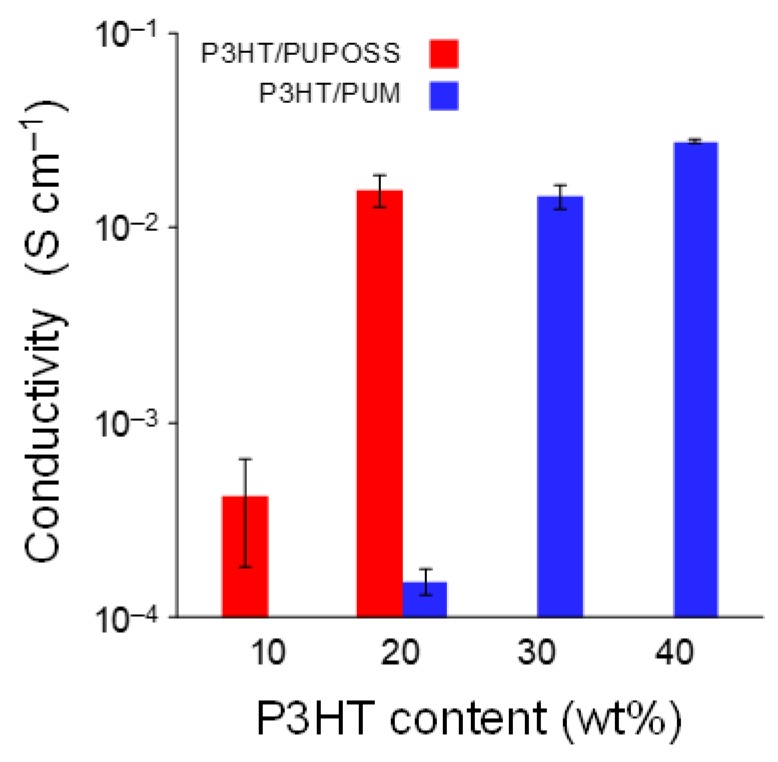
In-plane conductivities of P3HT/PUPOSS and P3HT/PUM.

**Figure 4:**
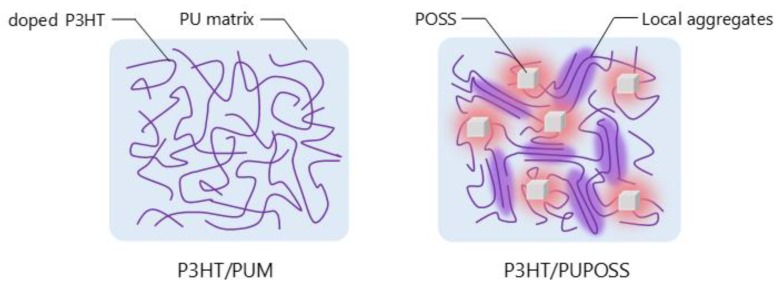
Illustration of the plausible mechanism regarding enhancement of conductivity by POSS.

In-plane electrical conductivity was measured with the doped P3HT containing hybrid films (Figure 3). The conductivities of the different surfaces, such as interfaces of air/hybrid film and hybrid film/PFA dish, were investigated. The conductivity of the surface from air/hybrid film interface was under a detection limit (less than 10^−6^ S cm^−1^), whereas that of the surface from the hybrid film/PFA dish was high. These results were in good agreement with the situation suggested by the SEM images. The ordered structures in aggregates of P3HT tended to be located at the interface of the hybrid film/PFA dish. Therefore, the conductivity was detectable only from the hybrid film/PFA dish. Thus, we evaluated electric conductivity only with this side. By changing the feed ratio of doped P3HT, significant behaviors were obtained. In both P3HT/PUPOSS and P3HT/PUM, the conductivities increased as the content of P3HT increased. However, in the same P3HT loading ratio, P3HT/PUPOSS had higher conductivity than P3HT/PUM. This means that POSS can play a significant role in the enhancement of electric conductivity in the film. According to the role of POSS in morphology as mentioned above, the mechanism can be proposed (Figure 4). From the SEM images and DSC measurements, it was suggested that POSS promoted aggregation of doped P3HT by enhancing hydrophobicity of matrices. In particular, it was implied that the ordered structures, where carrier transfer could efficiently proceed, could be formed in these aggregates [41,42,43]. From these data, it is proposed that POSS enhanced the carrier mobility, that is, conductivity through the facilitation of electric conductive paths. From the thermogravimetric analyses of the films, it was observed that all films had degradation temperatures (*T*_d_s) over 250 °C (Figure S12 and Table S1). From the investigation of influence of thermal annealing on electric conductivity, similar values were obtained from both films after annealing at 90 °C (Figure S13). In contrast, after annealing at 200 °C, conductivity decreased to out of the detection limit. This result supports that ordered structures in aggregates of P3HT originating from crystallization of alkyl side chains are responsible for efficient carrier transfer.

### 3.6. Influence of Strain on Conductivity

**Figure 5:**
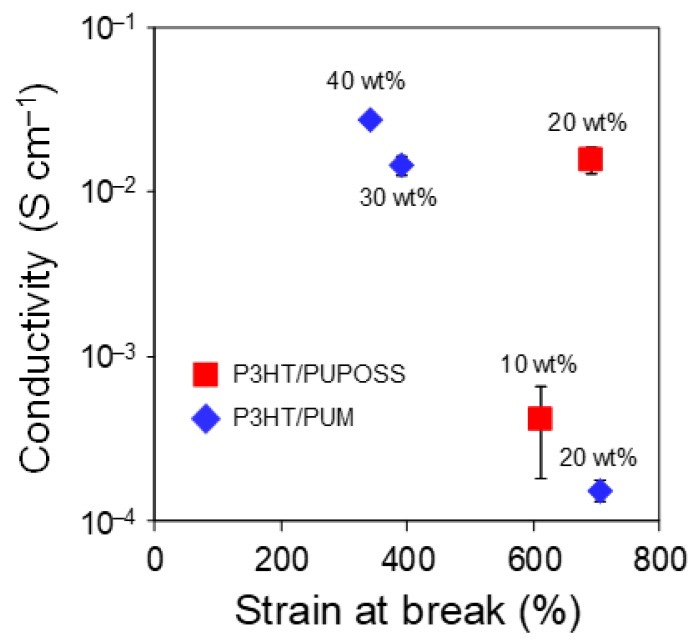
Correlations between conductivity and strain at break of P3HT/PUPOSS and P3HT/PUM at each amount of loaded doped P3HT.

**Figure 6:**
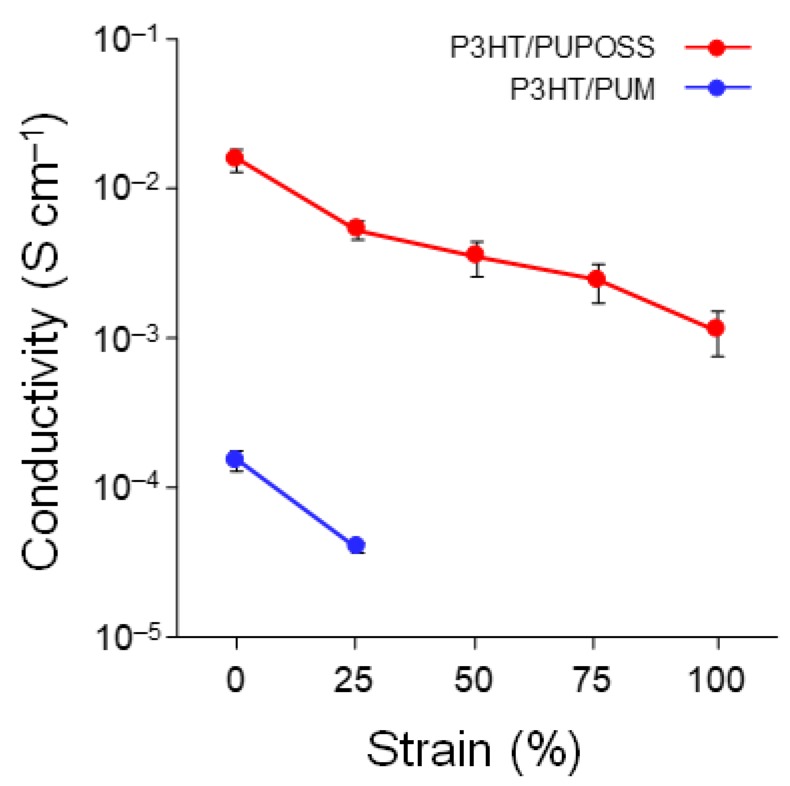
In-plane conductivities of the hybrid films with 20 wt % of doped P3HT under strain.

Finally, the influence of strain on conductivity of hybrid films was investigated. The film was cut into 1 cm × 2 cm and the in-plane conductivity was monitored up to 100% strain parallel to the conductive direction. Figure 5 shows the correlation with the strain value at the film breaking and conductivity with variable feed ratios of doped P3HT. By increasing the amount of doped P3HT in PUM, conductivity increased, while elasticity critically decreased. Fragility should be enhanced, resulting in lowing flexibility. Apparently, P3HT/PUPOSS exhibited high stretchability and conductivity. Owing to high conductivity even with a lower feed ratio of doped P3HT, both properties should be obtained. Next, conductivities of the films with 20 wt % of doped P3HT were monitored during stretching (Figure 6). The stretched films were fixed by clips on the quartz substrate on the measurement. Conductivity of the P3HT/PUM film was drastically lowered by stretching and dropped off under a detection limit immediately, meanwhile P3HT/PUPOSS kept conductivity at the relatively higher level even after stretching up to 100%. This result clearly demonstrates that conductive elastomers can be realized by employing POSS-based hybrid materials. It is likely that by the deformation of the materials, the orientation of the conductive network should be collapsed, resulting in loss of conductivity [74,75]. Enhancement of aggregation formation of doped P3HT by POSS should be responsible for the reinforcement of the network. As a result, critical losses of conductivity could be suppressed in P3HT/PUPOSS. We were able to obtain the relationship between conductivity and applied mechanical force (Figure S14). Our materials might be directly applicable as a coating or wearable sensor for tracing the distortion of the target surfaces.

## 4. Conclusions

We fabricated stretchable hybrids, P3HT/PUPOSS, consisting of POSS-capped PU and doped P3HT. The compatibility between PU and doped P3HT was decreased by the introduction of POSS due to the differences of polarity between polar doped P3HT and hydrophobic POSS. However, POSS promoted local ordered aggregates of doped P3HT, enhancing the electrical conductivity. As a result, P3HT/PUPOSS concurrently showed high stretchability and conductivity because it is possible to exhibit high conductivity even with small amount of doped P3HT. This simple method is expected to be applicable to a wide range of a combination of conjugated polymers and PU. It would be a conventional design strategy for the development of well-programmed stretchable electronics.

## Figures and Tables

**Table 1:** Mechanical properties of hybrid films.

Compounds	P3HT Content (wt %)	*E ^a^* (MPa)	*σ ^b^* (MPa)	*ε ^c^* (%)	*E′ ^d^* (MPa)	*E″ ^d^* (MPa)	tan*δ ^d^*	*T*_g_*^e^* (°C)
P3HT/PUPOSS	0	6.1	5.3	854	0.35	11	31	−15
	10	7.1	5.5	610	1.1	21	19	−33
	20	6.5	7.7	692	0.57	23	51	−8
	0	7.8	5.2	855	0.46	4.1	9.0	−28
	10	4.7	9.7	711	1.4	21	15	−39
P3HT/PUM	20	6.0	9.6	705	0.55	35	65	−2
	30	10	2.9	392	98	47	0.48	41
	40	35	3.4	340	69	45	0.66	34

^a^ Tensile modulus, ^b^ Tensile strength at break, ^c^ Strain at break, ^d^ Represented at 20 °C, ^e^ Glass transition temperature measured by DMA.

**Table 2:** Differential scanning calorimetry data of the films.

Compounds	P3HT content (wt %)	1st heating cycle				2nd heating cycle			
		*T*_1_ (°C)	*ΔH*_1_ (mJ mg^−1^)	*T*_2_ (°C)	*ΔH*_2_ (mJ mg^−1^)	*T*_1_ (°C)	*ΔH*_1_ (mJ mg^−1^)	*T*_2_ (°C)	*ΔH*_2_ (mJ mg^−1^)
P3HT/PUM	20	123	0.53	229	3.98	-	-	202	1.55
P3HT/PUPOSS	20	143	3.42	224	3.30	-	-	191	1.23
P3HT	-	-	-	227	12.0	-	-	227	12.5

## Supplementary Materials

The following are available online at https://www.mdpi.com/2073-4360/11/7/1195/s1, Figure S1: ^1^H NMR and expanded ^1^H NMR spectra of P3HT. Figure S2: Calculation of the POSS introduction rate. Figure S3: ^1^H NMR spectrum of HDO. Figure S4: ^1^H NMR spectrum of PTMG. Figure S5: Identification of chemical shifts of protons in ^1^H NMR spectrum of PUM. Figure S6: Identification of chemical shifts of protons in ^1^H NMR spectrum of PUPOSS. Figure S7: In-plane conductivity of F4-TCNQ doped P3HT films. Figure S8: Photographs of hybrid films. Figure S9: SEM images of the surface of hybrid films (interface of hybrid film/PFA dish). The amount of doped P3HT loaded in all films is 20 wt %. Figure S10: SEM images and elemental mapping of the cross section of hybrid films. The amount of doped P3HT loaded in all films is 20 wt %. Right side of the images is the interface of hybrid film/air. Figure S11: Storage and loss moduli of (a) P3HT/PUPOSS and (b) P3HT/PUM at each amount of loaded doped P3HT. Figure S12: TGA curves of (a) P3HT/PUPOSS and (b) P3HT/PUM at each amount of loaded doped P3HT. Figure S13: In-plane electrical conductivity of P3HT/PUPOSS and P3HT/PUM loading 20 wt % of P3HT before and after thermal annealing. Figure S14: The relationship between conductivity and applied mechanical forces of P3HT/PUPOSS loaded 20 wt % of doped P3HT. Table S1: TGA data of hybrid films.
