# Supplementary material for: Stretchable Conductive Hybrid Films Consisting of Cubic Silsesquioxane-capped Polyurethane and Poly(3-hexylthiophene)
Source: Polymers (Basel). 2019 Jul 17;11(7):1195. doi: 10.3390/polym11071195 (PMC6680475; doi:10.3390/polym11071195)
Supplement: Supplementary file 1 [file polymers-11-01195-s001.pdf]

Stretchable Conductive Hybrid Films Consisting of POSS-capped Polyurethane and Poly(3-hexylthiophene)

Keigo Kato, Masayuki Gon, Kazuo Tanaka* and Yoshiki Chujo

Department of Polymer Chemistry, Graduate School of Engineering, Kyoto University, Nishikyo-ku, Katsura, Kyoto 615-8510, Japan.

E-mail: tanaka@poly.synchem.kyoto-u.ac.jp

Contents:

Synthesis of PUM S–2

Synthesis of PUPOSS S–4

Expanded ^{1}H NMR of P3HT S–7

Calculation of POSS introduction rate in PUPOSS S–8

In-plane conductivity of doped P3HT films S–11

Photographs of hybrid films S–12

SEM images S–13

EDX images S–14

DMA data S–15

TGA data S–16

Thermal annealing effect S–17

Stress sensor S–17

Synthesis of PUM

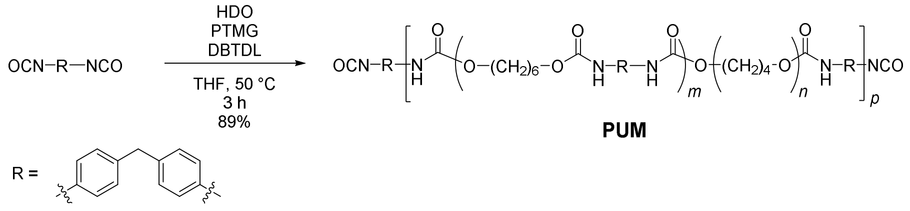

The mixture of HDO (0.14 g, 1.2 mmol), PTMG (1.2 g, 1.2 mmol), DBTDL (15 μL) and THF (10 mL) was placed in a round-bottom flask equipped with a magnetic stirring bar. Then MDI (0.75g, 3.0 mmol) in THF (2 mL) was added to the mixture via a syringe and the reaction was carried out at 50 °C for 3 h under Ar pressure. The resulting solution was poured into a beaker containing 150 mL of hexane, and white precipitates were afforded. The solvent was removed by filtration to afford PUM-MD (1.87 g, 89%). According to the ^{1}H NMR, the peak of hydroxyl group (2.28 ppm) disappeared. Therefore, it was presumed that all the alcohol was consumed and all ends of polyurethane chains were converted into isocyanate groups.

^{1}H NMR ($CDCl_3$, 400 MHz) δ 7.29−7.26 (brs, 4H, aryl-*H*), 7.08 (s, 4H, aryl-*H*), 6.76 (brs, 2H, -CON*H*-), 4.16−4.14 (m, 4H, -OCH_2-), 3.87 (s, 2H, -CH_2-), 3.82 (s, 2H, -OCH_2-), 3.41 (brs, 48H, -OCH_2-), 1.72 (m, 4H, -OCH$_2$CH_2-), 1.63−1.62 (m, 48H, -OCH$_2$CH_2-), 1.42 (brs, 4H, -OCH$_2$CH$_2$CH_2-) ppm; ^{13}C NMR ($CDCl_3$, 100 MHz) δ 136, 136, 130, 129, 119, 115, 70.7, 70.6, 70.2, 65.1, 40.6, 28.7, 26.5, 26.2, 25.9, 25.4 ppm.

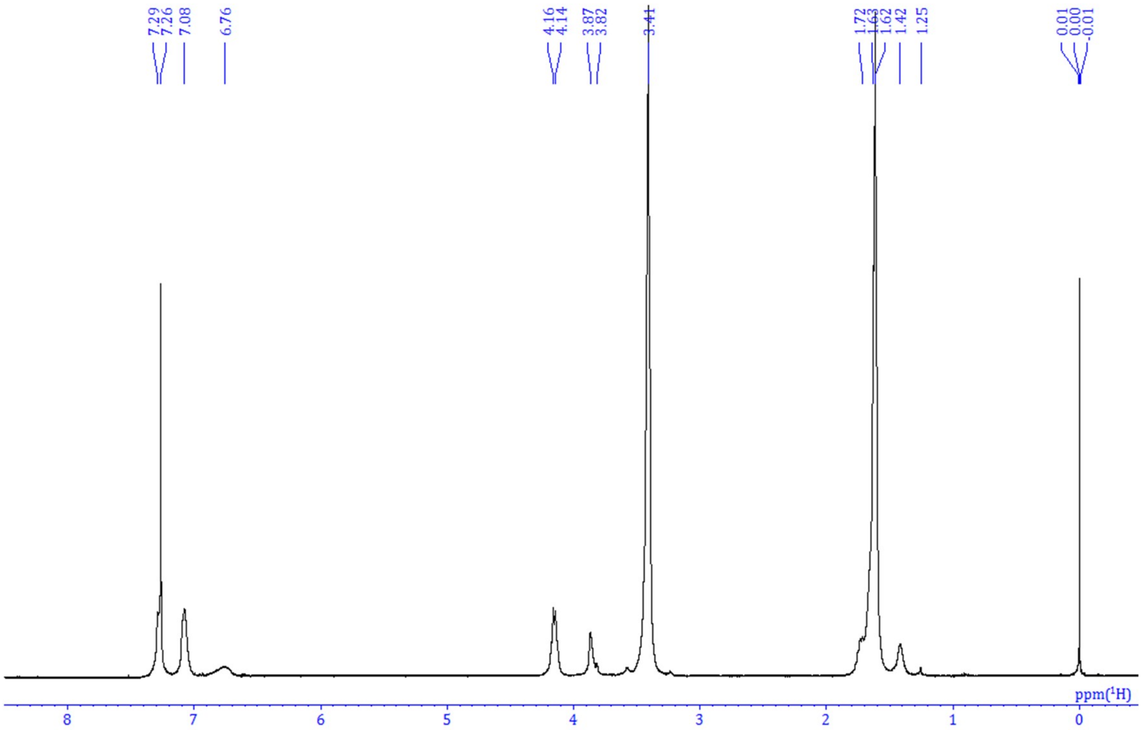

Chart S1. ^{1}H NMR spectrum of PUM.

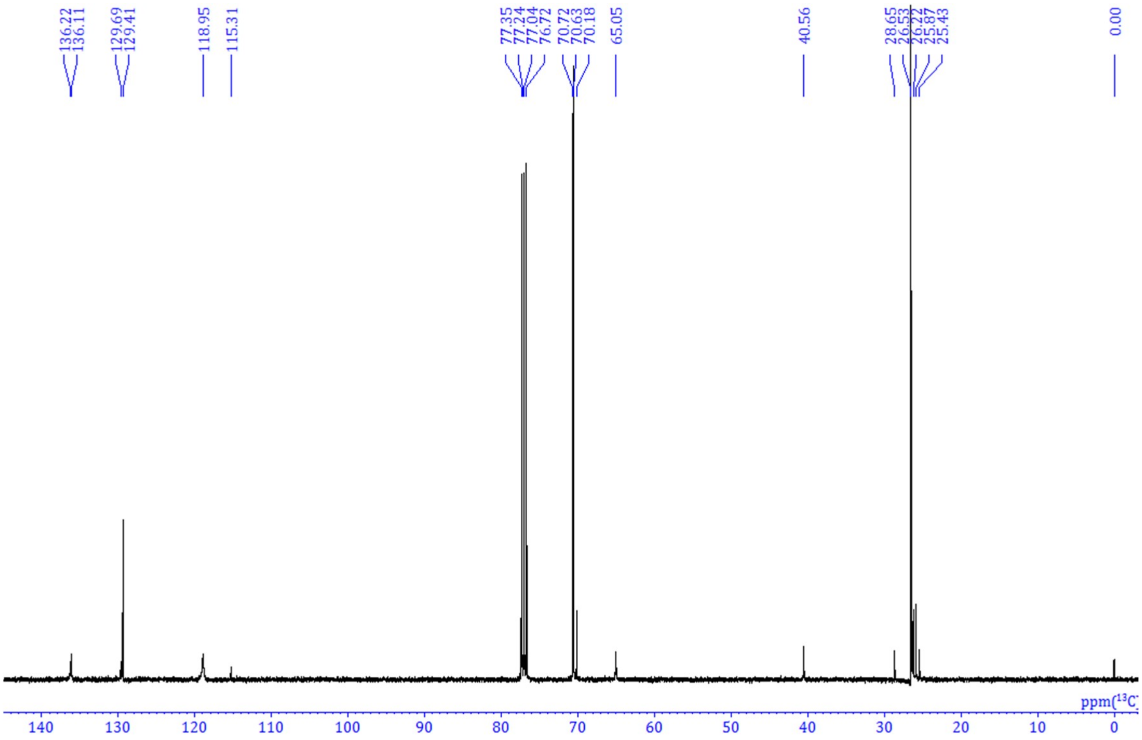

Chart S2. ^{13}C NMR spectrum of PUM.

Synthesis of PUPOSS

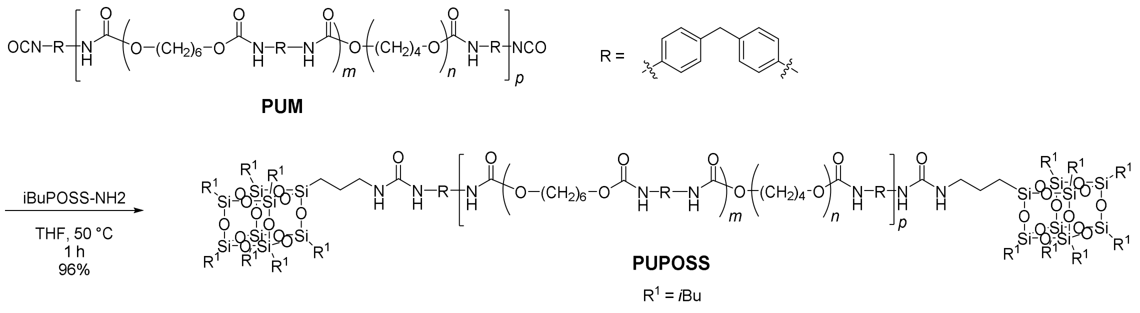

PUPOSS was obtained through the same procedure as PUM. Before reprecipitation by pouring into the hexane, iBuPOSS-NH_2 (0.13 g, 0.15 mmol) in THF (2 mL) or dodecylamine (69 μL) or propylamine (24 μL) was added to the mixture, respectively. Then the reaction was carried out at 50 °C for 1 h. The resulting solution were poured into a beaker containing 150 mL of hexane, white precipitates were afforded and the solvents were removed by filtration to afford PUPOSS (2.10 g, 89%).

^{1}H NMR ($CDCl_3$, 400 MHz) δ 7.27−7.26 (brs, 64H, aryl-*H*), 7.07 (brs, 64H, aryl-*H*), 6.74 (brs, 32H, -CON*H*-), 4.16−4.14 (brs, 32H, -OCH_2-), 3.86 (brs, 32H, -CH_2-), 3.41 (brs, 192H, -OCH_2-), 1.87−1.80 (m, 11H, -CH(CH_3)$_2$), 1.72 (brs, 32H, -OCH$_2$CH_2-), 1.61 (brs, 192H, -OCH$_2$CH_2-), 1.41 (brs, 32H, -OCH$_2$CH$_2$CH_2-), 0.96−0.93 (m, 63H, -CH_3), 0.61−0.59 (m, 21H, -CH_2-) ppm; ^{13}C NMR ($CDCl_3$, 100 MHz) δ 153, 136-136, 130−129, 119, 71.2, 70.7, 70.6, 70.2, 65.0, 40.5, 29.7, 28.6, 26.5, 26.2, 25.8, 25.7, 25.4, 23.8, 22.5 ppm; ^{29}Si NMR ($CDCl_3$, 80 MHz) δ –67.6, –67.6, –67.8 ppm.

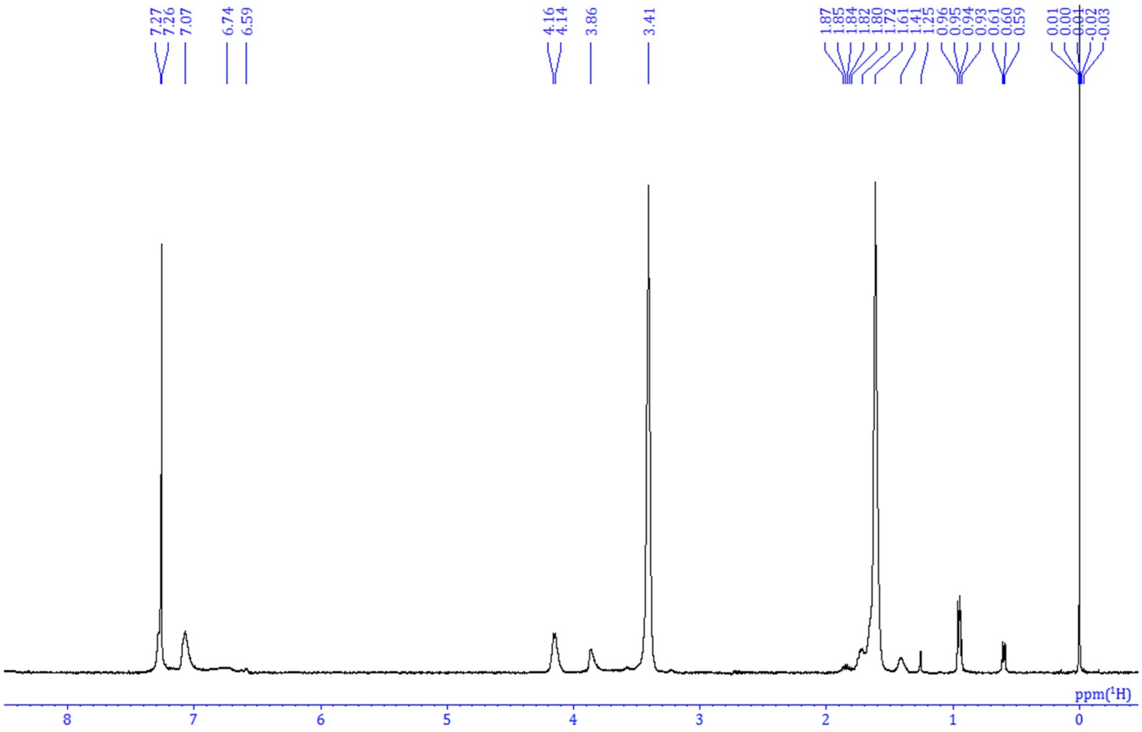

Chart S3. ^{1}H NMR spectrum of PUPOSS.

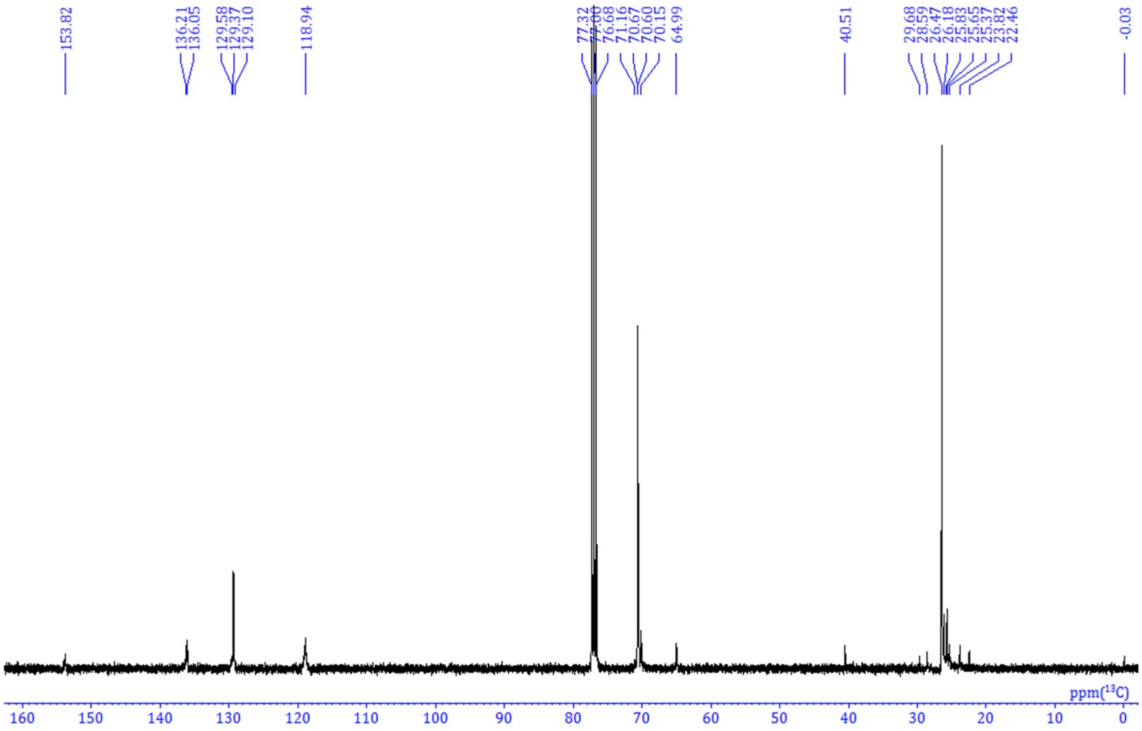

Chart S4. ^{13}C NMR spectrum of PUPOSS.

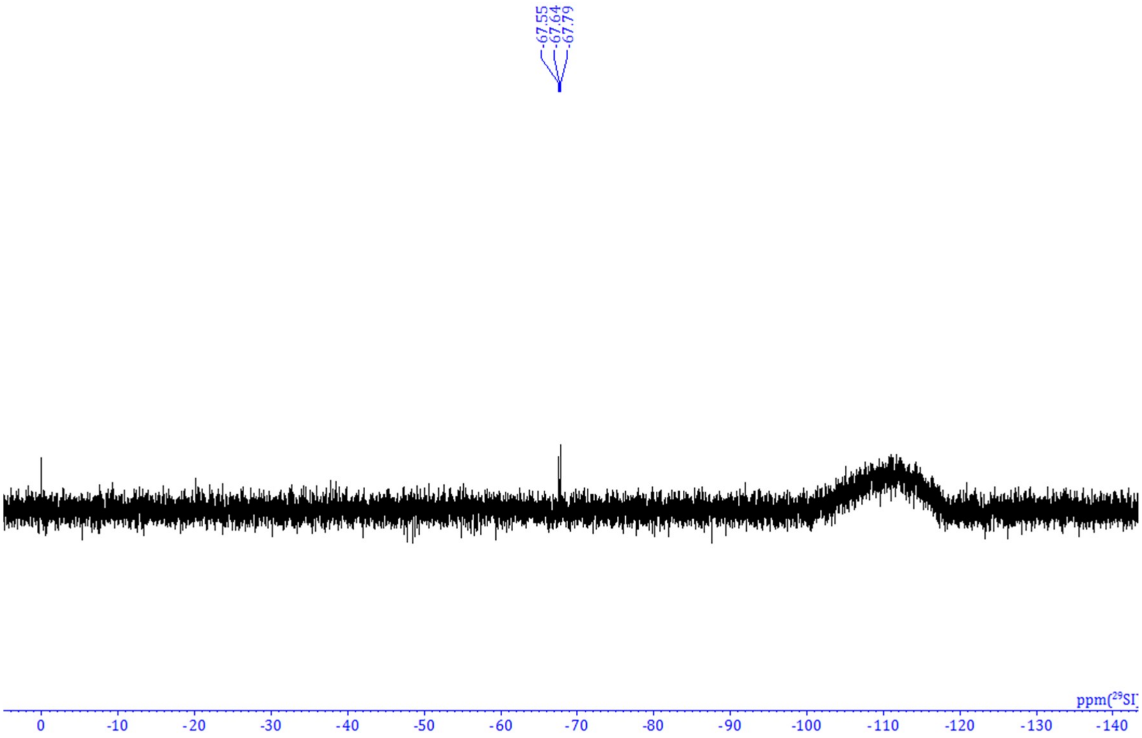

Chart S5. ^{29}Si NMR spectrum of PUPOSS.

^{1}H NMR and expanded ^{1}H NMR spectra of P3HT

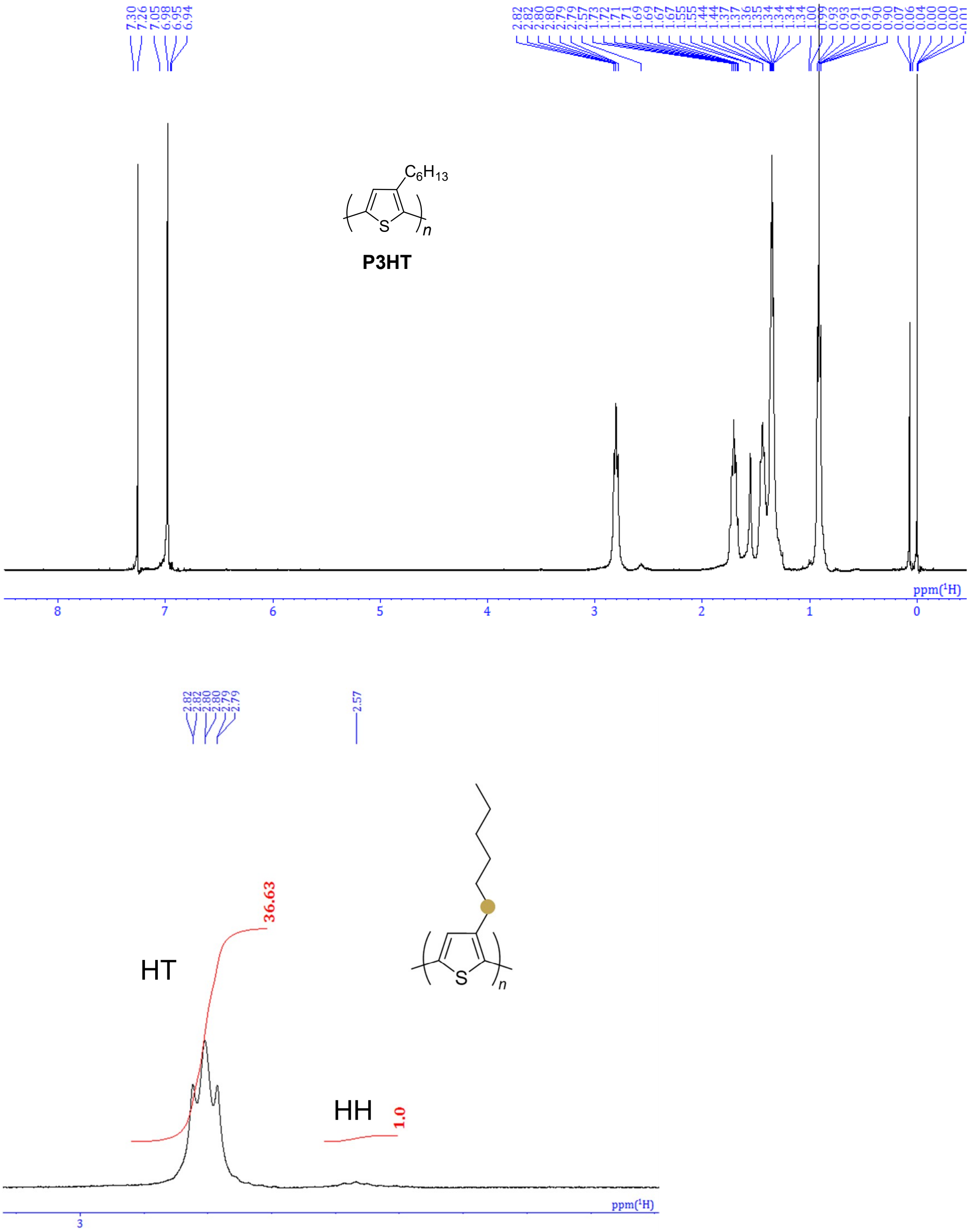

Figure S1. ^{1}H NMR and expanded ^{1}H NMR spectra of P3HT.

Calculation of POSS introduction rate in PUPOSS

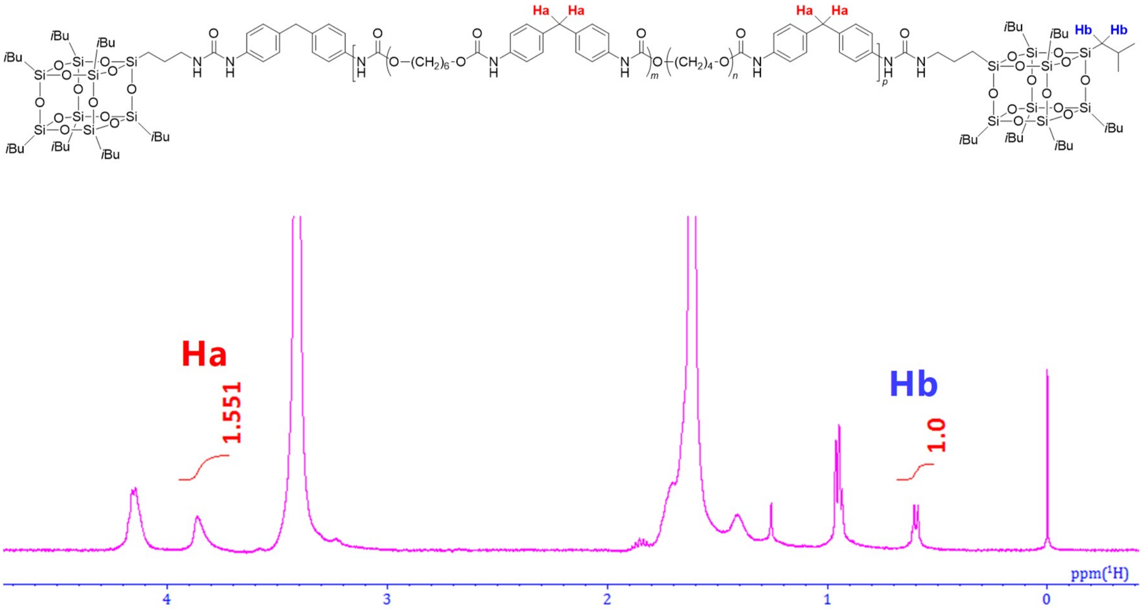

$M_n = 1.31 \times 10^4$

$M_{\text{repeating unit}} = 250 \times 2 + 118 \times 1 + 1000 \times 1$

$= 1618$

$M_n / M_{\text{repeating unit}} = 8.09$

$\text{Ha} : \text{Hb} = (8.09 \times 2 \times 2) : (2 \times \boldsymbol{x} \times 14)$

$= 1.55 : 1$

$\boldsymbol{x} = 0.75$

Figure S2. Calculation of the POSS introduction rate.

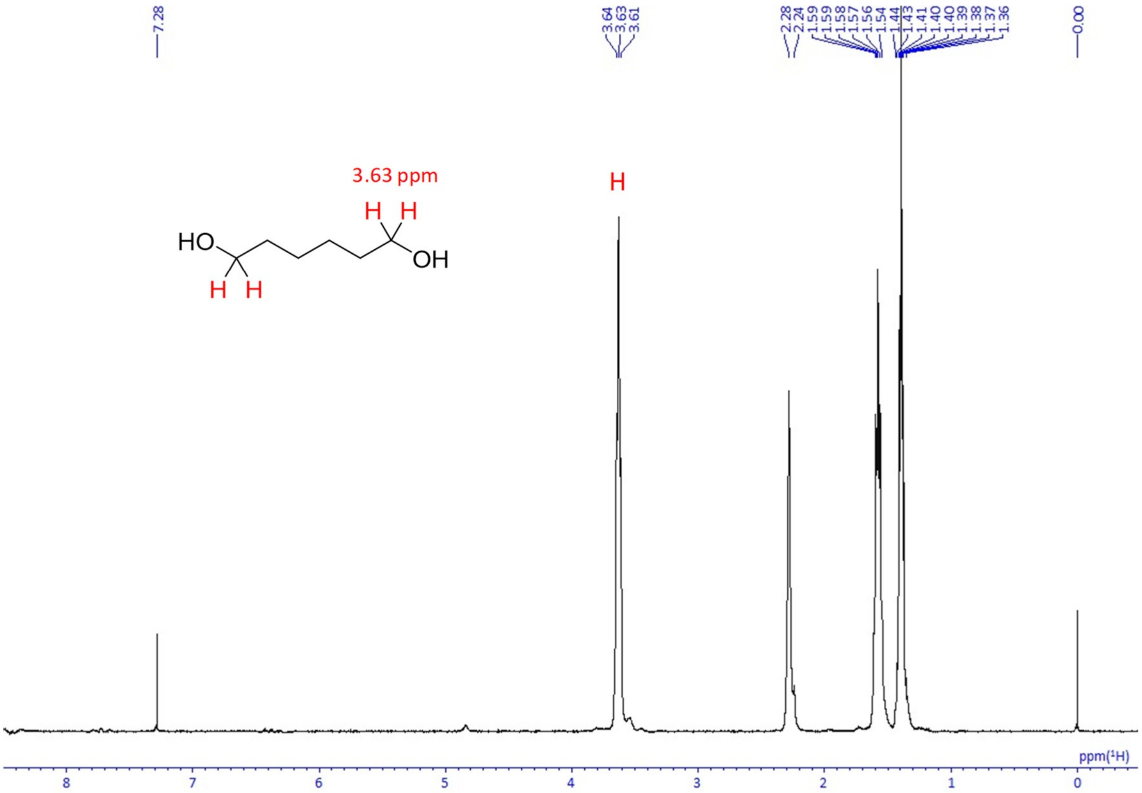

Figure S3. ^{1}H NMR spectrum of HDO.

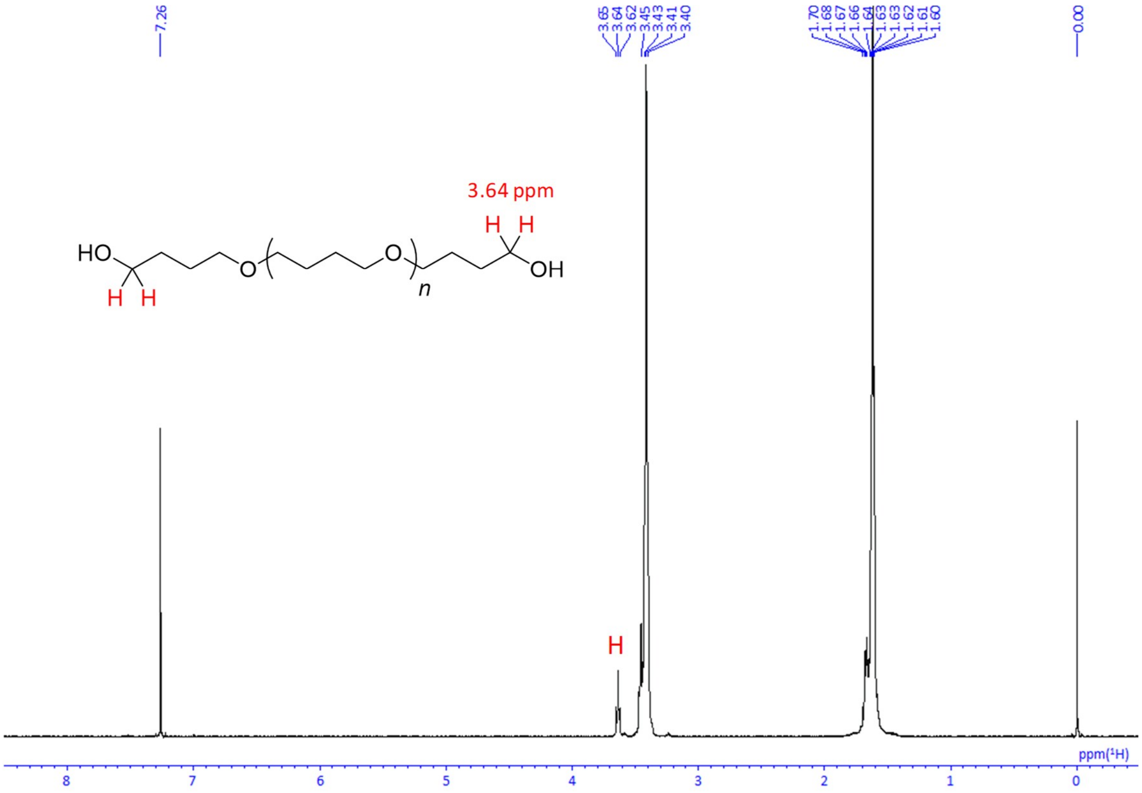

Figure S4. ^{1}H NMR spectrum of PTMG.

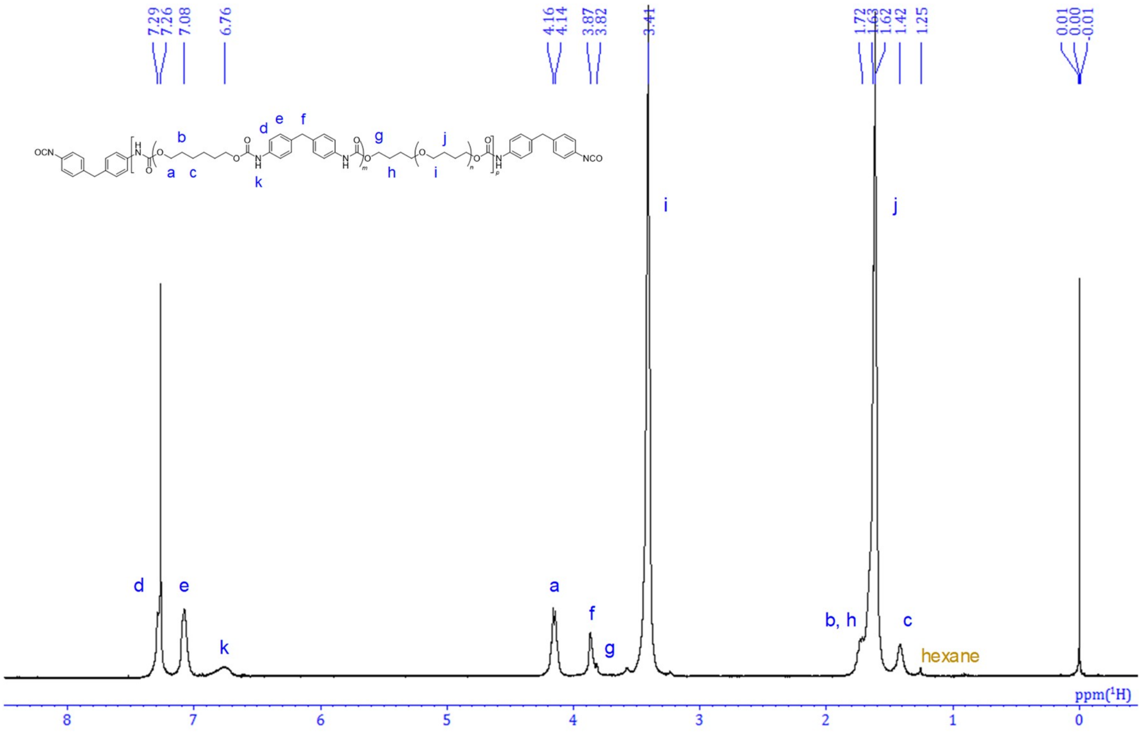

Figure S5. Identification of chemical shifts of protons in ^{1}H NMR spectrum of PUM.

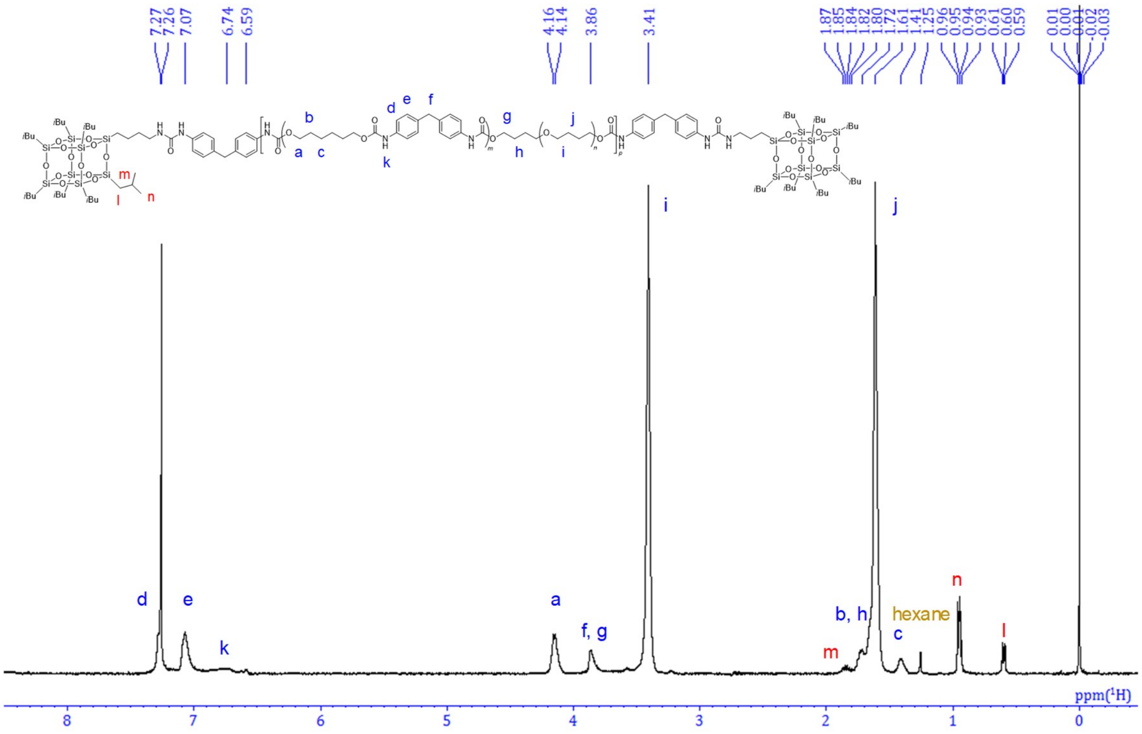

Figure S6. Identification of chemical shifts of protons in ^{1}H NMR spectrum of PUPOSS.

In-plane conductivity of doped P3HT films

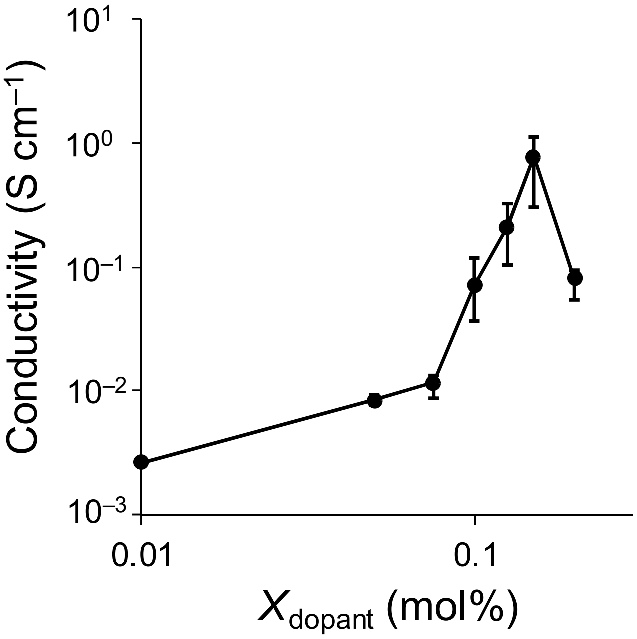

Figure S7. In-plane conductivity of F4-TCNQ doped P3HT films.

Photographs of hybrid films

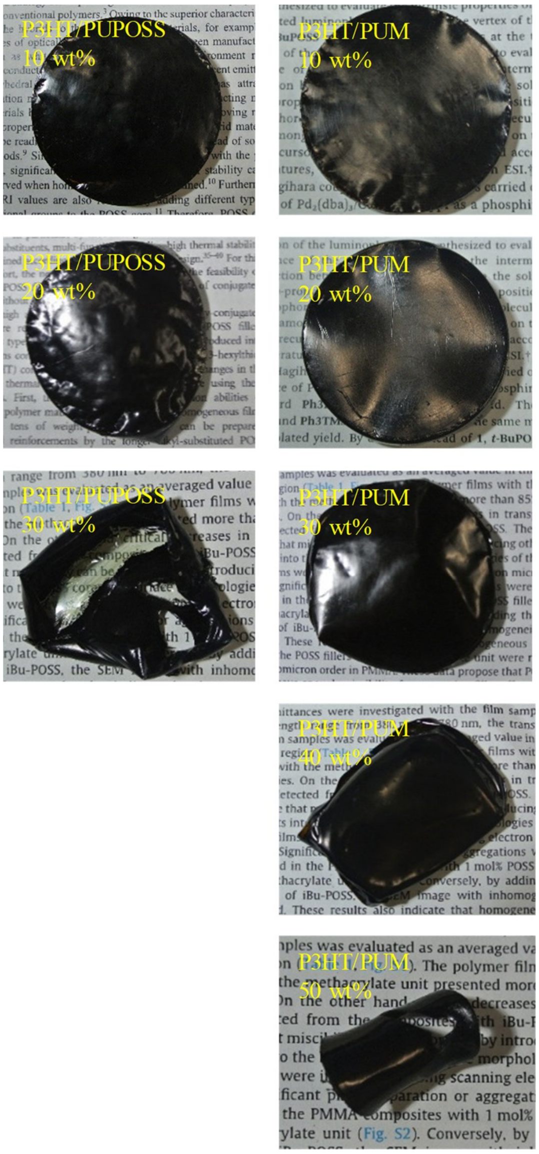

Figure S8. Photographs of hybrid films.

SEM images of hybrid films

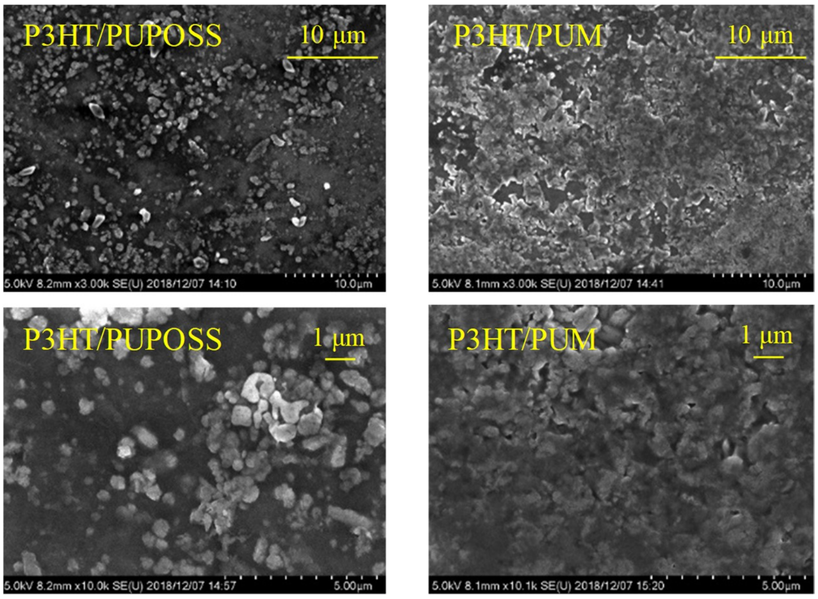

Figure S9. SEM images of the surface of hybrid films (interface of hybrid film/PFA dish). The amount of doped P3HT loaded in all films is 20 wt%.

EDX images of hybrid films

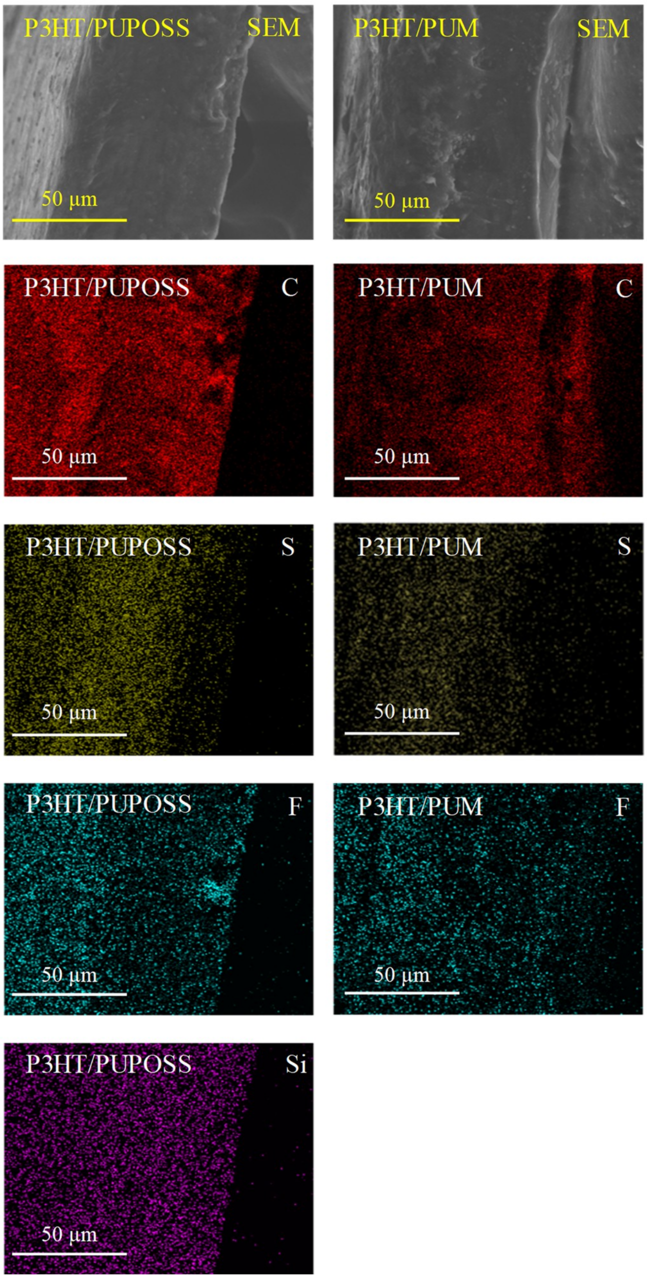

Figure S10. SEM images and elemental mapping of the cross section of hybrid films. The amount of doped P3HT loaded in all films is 20 wt%. Right side of the images is the interface of hybrid film/air.

DMA data

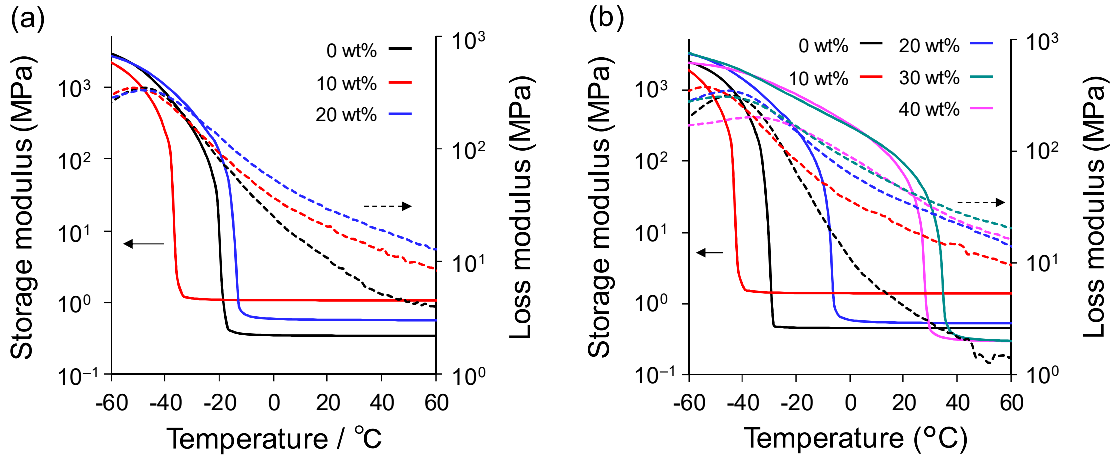

Figure S11. Storage and loss moduli of (a) P3HT/PUPOSS and (b) P3HT/PUM at each amount of loaded doped P3HT.

TGA data

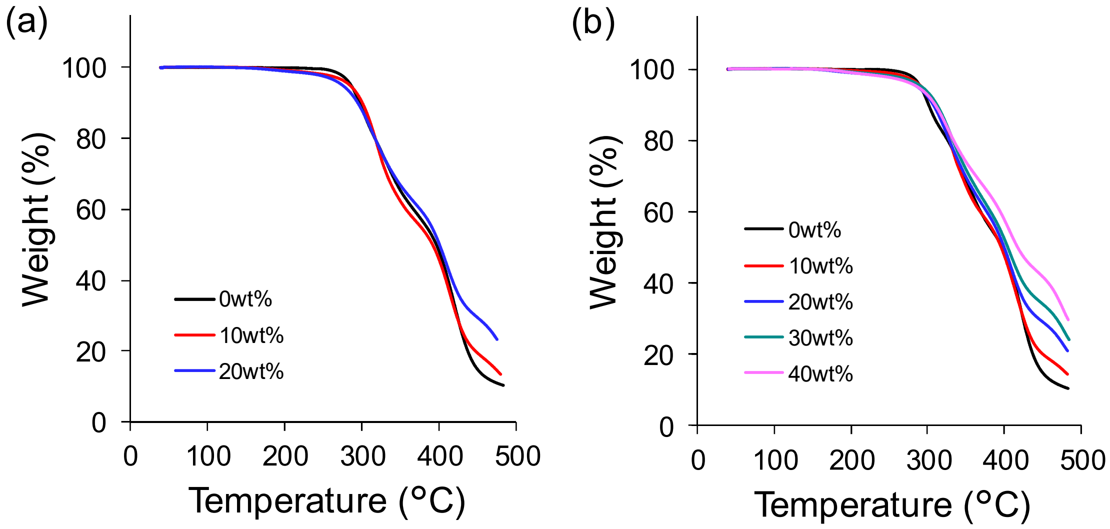

Figure S12. TGA curves of (a) P3HT/PUPOSS and (b) P3HT/PUM at each amount of loaded doped P3HT.

Table S1. TGA data of hybrid films

	P3HT content (wt%)	T_{d5} (°C)[a]	T_{d50} (°C)[b]
P3HT/PUPOSS	0	286	397
	10	285	392
	20	275	401
P3HT/PUM	0	291	397
	10	292	396
	20	287	400
	30	293	404
	40	287	417

[a] Determined at 5 wt% weight losses in the TGA curve. [b] Determined at 50 wt% weight losses in the TGA curve.

Thermal annealing effect

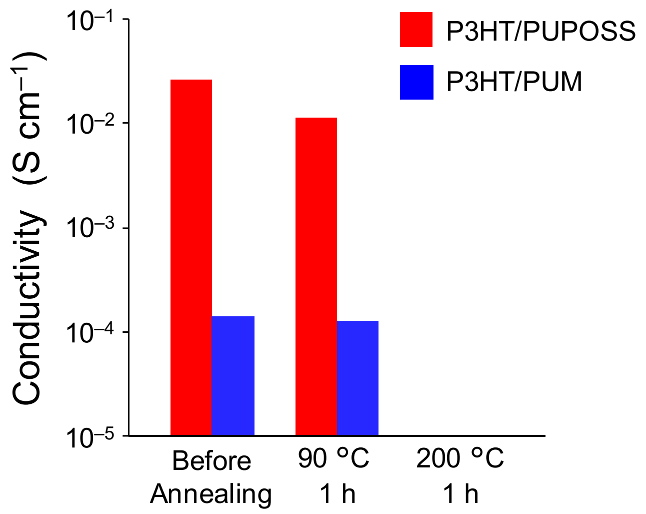

Figure S13. In-plane electrical conductivity of P3HT/PUPOSS and P3HT/PUM loading 20 wt% of P3HT before and after thermal annealing.

Stress sensor

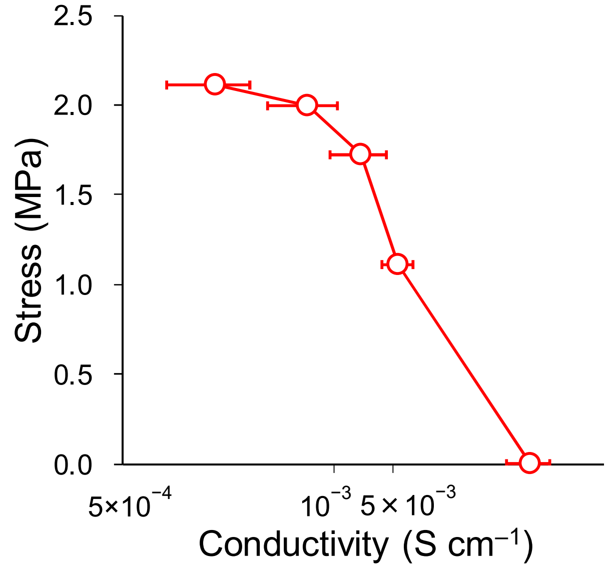

Figure S14. The relationship between conductivity and applied mechanical forces of P3HT/PUPOSS loaded 20 wt% of doped P3HT.
